# Facilitators and barriers to decision-making for hospital treatment among patients diagnosed with breast cancer in Dar es Salaam, Tanzania: A qualitative urban-based study

**DOI:** 10.1371/journal.pgph.0003366

**Published:** 2024-11-07

**Authors:** Pascal Mkaka Dominic, Masunga K. Iseselo, Raymond Athanas

**Affiliations:** 1 Department of Clinical Nursing, Muhimbili University of Health and Allied Sciences, Dar es Salaam, Tanzania; 2 Department of Bioethics and Health Professionalism, Muhimbili University of Health and Allied Sciences, Dar es Salaam, Tanzania; Universiti Malaya, MALAYSIA

## Abstract

**Background:**

Breast cancer is a major public health problem in both developed and developing countries and has become the second leading cause of death among women worldwide. The mortality may be related to delayed or inappropriate treatment decision-making among the diagnosed patients. Decision-making is an important determinant for successful treatment for patients diagnosed with breast cancer. In Tanzania, there is a lack of information in the context of facilitators and barriers to treatment decision-making after a breast cancer diagnosis. This study aimed to explore facilitators and barriers to treatment decision-making among cancer patients in Tanzania.

**Material and methods:**

A descriptive qualitative design was employed to explore the facilitators and barriers to treatment decision-making at Ocean Road Cancer Institute in Dar es Salaam. A purposive sampling technique was used to recruit fourteen female patients diagnosed with breast cancer. Data were collected through in-depth, semi-structured interviews, which were audio-recorded. A thematic approach was used to analyze the data.

**Findings:**

Two major themes emerged from the study findings, namely; facilitators to treatment decision-making such as patient understanding of treatment information, and healthcare providers’ support. Barriers to treatment decision-making include the cost of treatment, uncertainty about cancer treatment, and religious healing.

**Conclusion:**

This study found that practising decision‐making for hospital treatment remains a challenge for patients diagnosed with breast cancer. Patients’ understanding of treatment information and healthcare providers’ support are the main tools that can facilitate decision-making. Sensitization activities on breast cancer treatment in the community and coverage of insurance should be advocated to promote decision-making for hospital treatment.

## 1. Introduction

Breast cancer is a major public health problem in both developed and developing countries and has become the second leading cause of death among women worldwide [1]. Over 1.15 million women worldwide are diagnosed with breast cancer each year, and more than 500,000 of them face death [2]. Such high mortality may be related to delayed or inappropriate treatment decision-making among the diagnosed patients [3]. Furthermore, it is estimated that by 2035, two-thirds of new cancer diagnoses will occur in developing countries [4]. In sub-Saharan Africa, breast cancer is one of the leading causes of cancer death among women after cervical cancer [5]. In Tanzania, breast cancer is the second and most common leading cause of mortality after cervical cancer [6]. Most breast cancer patients in Tanzania present with advanced disease [7,8] making the treatment less effective and poor survival outcomes. Previous research in Tanzania found that there are more than 90% of female patients diagnosed with advanced disease stages (stages III and IV) [9]. With increasing emphasis on early detection and curative treatments, the specific needs of patients with breast cancer may be known and met.

Patients face several challenges when they are initially diagnosed with breast cancer. This may include a wide range of emotional experiences such as uncertainty, fear, helplessness, and/or anxiety [10]. Managing personal or private health information involves making decisions about whether or not to share data with others on an individual basis for several reasons, including bettering patient care and safety, patient autonomy, and patient voluntariness and preparedness to commence treatment [11]. Treatment decision-making is a multifactorial facet that involves many stakeholders. Studies in high-income countries reported that female patients preferred to share the decision-making with the physicians on the available treatment options to be implemented, regardless of whether the task is accomplished by themselves, with family, or with the physicians [11–13]. In this way, patients have greater involvement in decision-making, less decisional conflict, and greater satisfaction with the decision as compared to patients with less involvement [11]. Furthermore, the well-informed decision of patients with breast cancer gives patients a sense of control, increases hopefulness, allows them to confide in physicians what they fear, improves their ability to cope with suffering, and enables them to decide on all treatment options [14].

A guideline established by the Tanzania National Cancer Treatment Institute suggests that patients be involved in decision-making about the potential benefits and risks of all possible and available interventions and guided in making educated decisions [15]. These decisions may include whether or not to pursue chemotherapy, radiotherapy, surgery, hospital transfer, intensive care unit admission, or resuscitation [16]. Despite these guidelines, it has been reported that physicians fail to accurately identify patients’ preferences in treatment decision-making for breast cancer by perceiving that the patient’s desires are the same as the physicians’ in terms of treatment decision-making [13]. This hinders them from making appropriate decisions about their treatment. Lack of involvement in treatment decision-making causes patients to engage with traditional healers and go to spiritual groups [17,18] leading to poor treatment outcomes.

The provision of appropriate information is one of the facilitators for decision-making among patients diagnosed with breast cancer. Studies in developed countries revealed that patients with breast cancer are well-informed about their treatment [11,19,20]. The right of an individual to make decisions is one of the important aspects that can affect the treatment outcome of a patient with breast cancer. However, the patient’s decision on their treatment is hindered by paternalism, economic, family, and social-cultural factors [21,22]. In Tanzania, little is known about what facilitates or hinders treatment decision-making. The majority of the studies conducted have been focused on breast cancer from different perspectives such as breast cancer screening [23], truth-telling to cancer patients [24], experiences, and informational needs of women with metastatic breast cancer [25] rather than treatment decision-making. Therefore, this study aimed to explore facilitators and barriers to treatment decision-making among patients diagnosed with breast cancer in Dar es Salaam, Tanzania.

## 2. Materials and methods

### 2.1 Design and setting

A descriptive qualitative study design was used to ascertain the facilitators and barriers to treatment decision-making among patients with breast cancer. We used this design to gain insight from the participant’s perspectives. Also, treatment decision-making in this context has not been adequately explored [26]. Thus, this facilitated obtaining detailed information on how the patients make decisions related to the treatment. The designing and implementation of this study were performed according to the standard reporting of qualitative research (SRQR) (**S1 File**).

The study was conducted at the Ocean Road Cancer Institute (ORCI) in Dar es Salaam. It is located 200 meters from the Indian Ocean. The selection of the study area was made based on the fact that ORCI is a national referral centre for cancer treatment and provides care to over 5,400 new patients per year. There are 270 beds in the inpatient oncology unit, and 200–250 outpatients are attended daily. Most patients at this health facility report for diagnosis and treatment at the late stage of cancer development [27]. The diagnostic services provided include x-rays, ultrasounds, computed tomography (CT), bone scans, and fine needle aspiration biopsies with cytology. Treatment services provided at this facility are palliative care, nuclear medicine, chemotherapy, radiotherapy, and HIV care and treatment. Muhimbili National Hospital (MNH) and private hospitals offer additional services like histology, magnetic resonance imaging, and surgical care. Despite Tanzania’s limited insurance coverage, the country’s government and charitable partners subsidize radiation and diagnostic examinations at ORCI.

### 2.2 Participants, sampling methods and sample size

The study participants comprised female patients diagnosed with breast cancer, aged 18 years and above. Participants who were receiving treatment at the hospital participated in the study. Both outpatients and inpatients were involved. Participants who were critically ill were not included in the study as they could not endure with interview process. A purposive sampling was used to select the study participants. The researcher purposely sampled women diagnosed with breast cancer regardless of the stage and who could share their experiences on treatment decision-making. Potential participants who were coming for follow-up clinic and inpatients who met the criteria were approached for possible participation in the study. The research assistant approached eligible participants face to face and explained to them the need to be included in the study. Participants who voluntarily expressed their interest to participate were directed to contact the first author who then took their phone numbers for further communication and arrangement for appropriate day and time for the interview. A total of 14 participants were recruited based on information saturation. This is the point when new data did not bring or contribute additional insights to the research questions during collection and data analysis [28].

### 2.3 Data collection method and procedure

A face-to-face in-depth interview was used as a data collection method. The interview was conducted from 20 June to 15 July 2023 within the hospital premises of ORCI in a quiet and well-lit room for the comfort of both the participants and the interviewer. This provided a suitable environment that allowed the participant to speak freely without interference. A semi-structured interview guide was used to elicit questions. The contents of the interview guide were obtained through a literature review and consultation with experts in cancer treatment [29–32]. The interview guide consisted of questions about participants’ general views on hospital treatment, the information given before making the treatment decision, treatment decision-making involvement, and challenges encountered in the decision-making process. Each question was followed by several probing questions to get a deeper understanding of the topic in question(**S2 File**). Also, socio-demographic information was collected before starting the interview. The first author who had experience in qualitative research conducted all the interviews in Swahili language, and audio recorded. The interviewer had no prior relationship with the participants and was not involved in their recruitment. This facilitated freedom for participants to speak freely on the issues related to the treatment they were receiving. The first two interviews were used to pretest the tool. This was conducted with an open mind to determine the sequence and logical flow of the questions. Some questions were modified to increase the clarity. However, the contents of the interview guide were kept flexible in the subsequent participants to accommodate the emerging themes within our research objectives. The interviews ranged from 25–35 minutes. The thematic saturation was reached after 14 interviews.

### 2.4 Data processing and analysis

The analysis began once the first interview was conducted and continued throughout the data-gathering process. Following the completion of each interview, the first author began transcribing the data verbatim into written form. Then forward and back translation into the English language was performed by an experienced researcher as suggested by Chen and Boore[33] to ensure that the original meaning of the information from the participants is retained.

The thematic analysis as guided by Braun and Clark [34] was used to analyze the data. The first and second authors read the full transcripts and field notes independently to become familiar with the data and context. This process was important to find relevant information related to the research question. Deductive coding was used to analyse the data. The researchers developed codes based on research questions and then agreed on the codes that formed a codebook for subsequent analysis. The first two authors analysed the data. The first author coded manually by writing notes on the margins of the transcripts and using highlighters or coloured pens to indicate potential patterns in the text while the second author used Nvivo version 12(Developed by Lumivero (formerly QSR International)) to organize and analyze the data. In this case, important phrases, sentences, or paragraphs were coded. This process was repeated until all the data were coded. Inductive coding was also used for emerging information from the the data. This was important to ensure that all data were coded according to the set principles [35,36]. We then compared the codes developed independently by the two authors and the final codes were used to produce the themes. However, some codes were modified before further analysis. Then, the developed codes were combined to come up with general themes and some codes were combined to produce sub-themes of the main theme after noticing similarities, differences, and patterns among codes. The researchers reviewed the themes by renaming, merging, and removing some themes to ensure the final themes were mutually exclusive. Furthermore, the themes were compared with each other to identify the relationships among them. At this point, some themes were collapsed into each other, and others broke into themes and sub-themes and themes as illustrated by Byrne [37]. Thereafter, themes and sub-themes were arranged in a meaningful fashion with supporting quotes from the participants.

#### 2.4.1 Trustworthiness

We used Corbin & Strauss criteria [38] to establish the data’s rigour and reliability based on their credibility, dependability, conformability, and transferability. The researchers collected and analysed the data in the field for an extended period to increase credibility. Peer debriefing was employed to improve the dependability of the collected. Additionally, the first and second authors who were competent in qualitative research developed codes and categories independently and shared them for consensus. This process increased the code reliability. Sampling mode, questions development, the method of coding and category extraction and changes were documented to provide appropriate conformability.

### 2.5 Ethical consideration

Ethical clearance to conduct this study was obtained from Muhimbili University of Health and Allied Sciences (MUHAS), the Institutional Ethical Review Board with Ref. No.DA.282/298/01.C/1730. Permission to conduct this study was obtained from the Executive Director of the Ocean Road Cancer Institute before starting the data collection process. Furthermore, written informed consent outlining the purpose of the study, procedures, benefits, and risks of participating in the study was voluntarily obtained from each participant. They were also informed of the right to withdraw from the study and thus, they could quit at any point in time without giving any explanation. Participants were asked for consent about recording the conversation and all agreed to be audio-recorded during the interview. To maintain confidentiality, the audio was anonymously recorded without participants’ names.

## 3. Findings

### 3.1 Social-demographic characteristics of participants

This study involved a total of 14 female participants aged from 34 to 48 years. Among the participants recruited, three (3) were peasants, five (5) were housewives, three (3) were civil servants, and three (3) were self-employed. Also, eight (8) had a primary level of education, two (2) had a secondary level, one (1) held a certificate, one (1) held a diploma, one (1) held a bachelor’s degree, and one (1) held a master’s degree **(Table 1)**.

**Table 1:** Socio-demographic characteristics of participants.

Characteristics	Frequency	Percent (%)
**Age group (years**)	**Frequency**	**Percentage (%)**
25–35	3	22
36–45	9	64
46–55	2	14
**Educational level**		
Primary level	8	57
Form four	2	14
Certificate	1	7
Diploma	1	7
University degree and above	2	15
**Occupation**		
Civil servant	3	21.4
Self-employed	3	21.4
Peasant	3	21.4
Housewife	5	35.8
**Marital status**		
Married	6	43
Single	8	57

#### 3.1.1 Themes identified

Two themes and six sub-themes were generated in this study namely: facilitator for treatment decision-making, including patients’ understanding of treatment information, and healthcare providers’ support. Barriers to treatment decision-making include the cost of treatment, uncertainty about cancer treatment, traditional treatment, and religious healing practice **(Table 2).**

**Table 2:** Themes and sub-themes.

Themes	Sub-themes
Facilitators to treatment decision-making	1. Patients’ understanding of informed consent 2. Healthcare providers’ support
Barriers to treatment decision making	1. Cost of treatment 2. Uncertainty about recovering from cancer 3. Traditional treatments 4. Religious practice

### 3.2 Theme one: Facilitators to treatment decision-making

#### 3.2.1 Patients’ understanding of treatment information

Participants revealed that a consent form was necessary when involving patients in treatment decision-making. They expressed that understanding of the required information helped them in making informed decisions about cancer treatment. They stated that the attending clinician discussed the essence of the diagnosis and the required treatment on the first day before undergoing any medical procedure. This step was important to increase awareness of the benefits, risks, and side effects of the medicine they would receive in case they continue with hospital treatment. They asserted that they were required to sign the form as evidence of understanding of the information as expressed by one of the participants:

*“…from the beginning, before starting the treatment, there was a form if I was ready to be treated. To agree to this treatment, you must sign the form as a sign of understanding of the information provided. So I signed for my will that I am already after having fully informed.” (PT #1).*

Furthermore, participants commented that the availability of treatment options enabled them to ask different questions and get clarifications about treatment and medication. They commented that the face-to-face method of delivering information to them was convenient and facilitated them to make faster decisions about their treatment options available without making unnecessary delays. Hence, the provision of information was an important facilitator for them to make decisions about their treatment as elaborated in the following statement:

*"…I became free to decide on my treatment after being told the type of treatment available by my doctor, including the benefits and harms of those drugs. No one interfered with me, even at the hospital, so I decided to get treatment myself.” (PT#5).*

Additionally, participants expressed that they were involved in treatment decision-making and the fact that healthcare providers allowed them to decide according to their understanding of the information provided. Involvement in decision-making increased their autonomy and their decision was not influenced by anyone else except the information provided in the health facility. They expressed to have informed about the treatment alternatives including risks, medication side effects, and benefits of the treatment, before they undergo any medical procedures. One participant explained this:

*“…the doctor told me that I would lose my hair, I would get some infections, and my white blood cells would drop. After initiation of treatment, experienced such problems such problems. Since I had prior information, I had to persevere with the treatment side effects” (PT#2).*

#### 3.2.2 Healthcare providers’ support

Participants stated that they were motivated to seek treatment at the hospital because the services offered there allowed them to feel secure during care. They asserted that the good rapport established by the healthcare providers facilitated their acquired hospital treatment. They added that healthcare providers’ communication and counselling skills contributed to their decision to start hospital treatment. This was verbalized by participants who had visited different health facilities before joining the current hospital as stated by one of the participants below:

*“…I thank God that since I have started being treated here, I have not been subjected to any cruelty by healthcare providers. What they tell me to do is encouraging. Even though I have a difficult economic situation to the point that I am unable to follow the medicine as required, the services they offered me here are good that motivated me to continue with hospital treatment.” (PT#12).*

Some participants explained that the healthcare providers encouraged them when they felt desperate. They expressed that the treatment regime was difficult to follow and adhered to its complexity. Side effects of treatment increased their likelihood of dropping out of hospital treatment and aspired to alternative treatment services. However, healthcare providers were closely monitored and encouraged to continue with treatment as per the treatment regimen. Also, they stated that the healthcare providers provided attractive arguments during counselling sessions that facilitated embracing hospital treatment and procedures. One of the participants commented:

*“…at this hospital, I met the Doctor who has been pursuing me and talking to me well and nicely about my treatment by telling me that this disease has a treatment I should not have to worry I should pay attention to my treatment and they have given me advice for a while but I understood better than before I did not have a complete understanding.” (PT14).*

### 3.3 Theme two: Barriers to decision-making for hospital treatment

#### 3.3.1 Cost of treatment

Financial problems were the most reported barrier to making decisions for hospital treatment initiation. Participants described the high cost of chemotherapy and radiation as a significant obstacle. They described that treatment was expensive and required a lot of money to undergo those chemotherapies and radiations. This was an obstacle for them to make early and timely decisions for treatment at the hospital as verbalized below:

*“…I don’t know what to do with this disease, the treatment is very expensive and requires a lot of money and I was told that I will be given six chemotherapy treatments, and each one costs 200000/ = shillings* [more than 80 USD] *now ask yourself about the situation of a Tanzanian who does not have a job.” (PT#11).*

Further, the participants stated that when approached by healthcare professionals regarding their medical treatment, they discovered that it would cost a lot of money and that they did not have a good financial situation. As a result, they sometimes postpone treatments because of lack of funds, the patients might only receive painkillers as a treatment as a results delayed making the proper decision. One participant said that:

*“…If I were to get treatment on time, I would have finished the rounds of chemotherapy and radiation; even my breast could have been removed, but money is very difficult; the challenge of making money is very difficult, and I mean, I don’t even get treatment on time." (PT#8).*

Other participants said it was very challenging to deal with the issue of income. They said that having cancer and being constrained by financial problems made it difficult to receive timely treatment. Instead of concentrating solely on seeking therapy, they had to work first to get money to cater for the treatment cost which affects the patient’s choice of treatment, as in the statement below:

*“…financially, is a challenge, I don’t have a job right now, which means you need to wait for other people to help you, so I had a very, very big challenge. For example, I have skipped two circles of chemotherapy and I have not received medicine because chemotherapy is very expensive and I have no money.” (PT#12).*

#### 3.3.2 Uncertainty about cancer treatment

Participants expressed that they feared and worried about cancer diagnosed. They thought that they would die soon. They verbalized that they started to panic and display dissatisfaction with their future life due to the factor that cancer was incurable. They described that had a lot of thoughts in their minds regarding their situation of being diagnosed with breast cancer. This situation hindered them from making proper decisions, thus delaying to start of hospital treatment. One participant lamented:

*“…I was uncertain about the treatment outcome, it is not a secret, that our income is little, and thinking of this disease in terms of the treatment is very challenging and possibly not curable. So, if you think of the possibility of cure, it is a challenge” (PT#10).*

Also, participants reported that they were confused after learning that they had breast cancer. They expressed that they were uncertain if they would be cured in the hospital and hence thinking of the cost of investing in treatment. This thinking pattern hindered them from making proper treatment decisions. They struggled to accept the situation for a long time and tried to figure out what was causing them to suffer, hence delaying seeking timely treatment. They also added that they reached a point when they did not want to disclose their diagnosis except to their spouse as in the description by the participant below. One participant said:

*“…just saying the word cancer is a very big name, and I am scared. That, will I recover from cancer one day? will this disease leave me safe? I am thinking a lot because there are many opinions in our society concerning cancer. At first, I hid it I never told anyone that I was sick but later I only informed my close people, such as my husband and my children."(PT#1).*

#### 3.3.3 Traditional treatment

Participants admitted that after receiving a cancer diagnosis, they initially considered looking for traditional healers who would prescribe them traditional medicine to take for several days before beginning hospital treatment. However, they said that was not helpful as they were not cured. They expressed that after realizing that their assumptions were incorrect, they sought medical attention when they discovered that they were still ill. The participants explained this:

*“…as a human being, I also looked at the other side, I went to natural remedies, and I was convinced to come here after I got answers that I have cancer, I said there may be another alternative, and I can be helped to solve the problem. I used Maasai medicines to drink, but I did not see any difference, so I decided to continue with hospital treatment.” (PT#3).*

In addition, participants reported using a lot of traditional medicine as prescribed by their traditional healers. This practice was described as wasting a lot of time managing such medications without even getting cured. Their condition worsened. Once they decided to go to the hospital to continue with the treatment, they discovered it was already too late and the disease condition had reached stage four. Below is what one of the participants said regarding this:

*“…I have used a lot of medicines, first I used the leaves of msatakafari* (local herbs*). They used to blend it for me and used it for three months, then I stopped using it. I also used turmeric and I used some flowers like this, the roots of which I used up by boiling and drinking a quarter cup every day, then I used some flowers like a lover.” (PT#8).*

Participants stated that their family members and relatives influenced them to follow traditional healers by influencing them to stop taking their hospital treatments and medications. They added that after taking the prescribed medication repeatedly from different traditional healers, still they saw no progress, hence they became sicker than before, causing a lot of pain, and their condition became worse. One participant elaborates that:

*“…it was only local medicine that I was given and I used them for some time while believing that I would recover and my condition would improve but unfortunately it was not as I expected that I would recover, as my relatives were persuading me that if I take the medicines of traditional healers, I will recover.” (PT#9).*

#### 3.3.4 Religious healing practice

Participants admitted that they frequently attended prayer camps in the hopes of being healed, but the results remained the same or worsened. They verbalized that repeated attendance to the spiritual leaders resulted in to delay in seeking medical treatment. Also, participants reported that, patients who had initially started hospital treatment did not continue with hospital treatment due to believing that the healing power from spiritual leaders could heal and cure breast cancer. Some of them continued to believe that they were getting better though they were receiving treatment in hospitals even though they were still ill. But for others, their faith did not change towards hospital treatment. One participant made the following statement:

*“…I have gone to SAFINA prayer camp and I have taken all the healing symbols like salt and oil. I used all the indicators, which is why I believe that I have recovered. But when I went to the test I knew that I was recovered but was not cured spiritually that is why I am being treated here for now.” (PT#14).*

Moreover, the participants stated that after receiving a diagnosis, they had no other choice but to return to the Almighty God and attend various religious gatherings because they believe that only God has the power to heal people, and this is because after hearing testimonies from others, they believed that they would also be healed. For this reason, they were not concerned with medical treatment during this time and hence delayed in initiating hospital treatment. One participant reported this:

*“…I used the religious method groups by attending various religious conferences and fasting a lot so that I could get healing. Based on the testimonies I was hearing from the radios, I also believed that if I went there I would be healed too. But I wonder if the results are the same.” (PT#9).*

## 4. Discussions

In the current study, patient understanding of treatment information and healthcare providers’ support are the facilitators for treatment decision-making. Barriers to treatment decision-making include the cost of treatment, uncertainty about cancer treatment, traditional treatment, and religious healing practice.

Patient’s understanding of treatment information is useful, especially when involving patients in treatment decision-making at the beginning of their treatment. This is because the information provided enables patients to become aware of the benefits, risks, and side effects of medication [39,40]. Also, as part of demonstrating patients’ autonomy, a written agreement is required for all treatment options, including interventions, chemotherapy, radiotherapy, and screening departments [39]. In our context, understanding of the information is facilitated by the belief that patients trust healthcare providers. The findings are in line with the Tanzania National Cancer Treatment guideline, which suggests that patients be assisted in making informed decisions based on this information and a knowledge of what is important to them as individuals by providing them with information about the potential positive aspects and dangers of all accessible and possible interventions before undergoing any medical procedures [15].

Healthcare providers’ support as reported in our study indicates that a good relationship between the healthcare provider and the newly diagnosed breast cancer patient is the main motivator for decision making to start treatment in the health facility. This can be supported by the assertion that good communication with patients has an impact on diagnostic accuracy, aiding recall of information provided and treatment adherence [41]. Our findings indicate that healthcare providers and patients had positive interactions, allowing them to understand their medical information and choose the treatment they wanted. Similar findings were reported in the Netherlands in which oncologists had a positive influence on general decision-making for women diagnosed with breast cancer to initiate treatment or proceed with treatment [42]. According to another study conducted in Ghana, women have been involved in various stages of treatment decision-making, interacting with informal networks and specialists about the need for chemotherapy [11]. This is an important aspect that health professionals need to practice in patients with breast cancer in particular. Unlike our study, the systematic review of patients with cancer and health professionals’ interaction revealed negative experiences on preferences regarding communication and the type of personal support needed were ignored [43] and thus recommended continuous education and professional development for healthcare professionals regarding their verbal and non-verbal communication, empathy, active listening, and collaboration skills. Our findings emphasized the importance of timing, honesty, and clarity of information regarding health care provided during the counselling session. This facilitated our study population to adhere to and continue with the treatment regime despite the side effects of chemotherapy.

The findings of this study also revealed that cancer treatment is costly and affects the decision-making for hospital treatment. Although cancer patients at government hospitals have to pay for screening, if diagnosed, treatment is free [8]. This implies that patients need to pay for some procedures, particularly in the initial stage of diagnosis. The out-of-pocket cost hinders them from seeking hospital treatment in our setting and other low-income countries. The lack of a health insurance scheme compounded by poor income makes the situation difficult for our study participants to seek hospital treatment. Similar findings have been reported in Nigeria [44,45] United States [46] and Ethiopia [47]. Our findings are also supported by a study in Uganda, which found that many breast cancer patients stop their treatments due to financial constraints and difficulty navigating the healthcare system [48]. This implies that patients with breast cancer are unable to afford treatment costs. As observed by Ezeome and Anarado [49], patients who must pay for breast cancer treatment out of their pockets are more likely to seek alternative ways to manage their breast cancers. In line with this, a similar study conducted in Tanzania reported that many participants expressed concerns about the financial impacts of cancer treatment, including medication and treatment being expensive, and that they were unable to receive full treatment due to a lack of funds [50]. As one of the strategies to guarantee health for all, the Tanzanian government has adopted universal health coverage (UHC) that guarantees that no one is denied access to necessary medical treatment because of their inability to pay for it [51]. In this context, Tanzania’s Health Financing Strategy (HFS), can play a key role in advancing UHC inclusive of the poor. This can benefit cancer patients who need costly treatment such as chemotherapy and radiotherapy.

Concerning uncertainty of cancer treatment expressed by our study participants can be described by the stigma attached to cancer diagnosis. The belief that cancer is incurable contributes to the perceived stigma among patients diagnosed with breast cancer [52–54]. The uncertainty of cancer treatment might have been caused by low awareness of the condition as highlighted in other studies elsewhere in Tanzania [55,56]. Sensitization activities and education to raise awareness on cancer-related issues should be promoted among the study population. This can facilitate decision-making for hospital treatment. The current findings are in line with those reported by Masika et al [48] in the same setting that described the shock and sadness of being given a cancer diagnosis and the likely outcome of breast cancer. While participants had worries, stress, and depression that impaired them in making appropriate treatment decision-making regarding treatment, this study highlights the need to create awareness that will dispel such uncertainty regarding treatment options among women diagnosed with breast cancer in Tanzania.

Herbal medicine is another barrier to deciding on hospital treatment in our study population. Several reasons can be attributed to this practice including recommendations from family and friends, perceived benefit, compatibility, and healer credibility [57]. Also, the tendency may have been influenced by the patients’ perception that traditional medicines are safer and more effective and the lack of cancer services in other parts of the country [58,59]. However, this practice is more likely to cause delayed treatment hence the patient present in the hospital at the advanced stage of cancer disease [59]. The findings in this study are supported by a review study from Sub-Saharan countries which reported that social beliefs affect breast cancer as believed to the the results of punishment by ancestors leading to seeking traditional services [60]. Health promotion focused on raising awareness of hospital treatment should be advocated.

The role of religious healers as a barrier to decision-making for hospital treatment can be accounted for by the fact that diagnosis of breast cancer makes patients feel vulnerable, anguished, pessimistic, hopeless, and anxious about impending death. Given the pain and the uncertainty, something transcending makes patients fight for life through visiting prayer camps [61,62] thus delaying hospital treatment. Comparable findings have been reported in other countries such UK [63] and Brazil [64,65]. The influence of religious healing in Tanzania has also been reported to affect treatment outcomes of other chronic diseases such as HIV [66]. The healthcare system needs to look for mechanisms to involve religious leaders as part of psychosocial support for women with breast cancer. This is an interesting future research area on how to integrate hospital treatment and religious services for patients diagnosed with breast cancer.

## 5. Limitation

This study can not be without limitations. As the study was conducted in urban areas, the findings may not be transferable to people from rural settings. Moreover, socially desirable responses may have occurred due to face-to-face interviews during data collection. Finally, since we were interested in the treatment decision-making for female patients with breast cancer, we only used a single data collection method rather than multiple data sources which would have allowed for the triangulation of the information provided. In addition, some thematic areas might have not provided enough information during the interview due to fear of the environment as the interview occurs in the hospital setting. However, the use of probes and the skills of the interviewer helped to elicit information that makes the findings of the study valuable for the study population.

## 6. Conclusions

This study found that practising decision‐making for hospital treatment remains a challenge for patients diagnosed with breast cancer. Patients’ understanding of treatment information and healthcare providers’ support are the main tools that can facilitate decision-making. Coverage of health insurance can help with the problems of costly cancer treatment. Sensitization activities and education to create awareness of breast cancer in the community should be advocated to promote decision-making for hospital treatment.

## Supporting information

S1 File SRQR checklist. (DOCX)

S2 File Interview guide. (DOCX)
