# Supplementary material for: Facilitators and barriers to decision-making for hospital treatment among patients diagnosed with breast cancer in Dar es Salaam, Tanzania: A qualitative urban-based study
Source: PLOS Glob Public Health. 2024 Nov 7;4(11):e0003366. doi: 10.1371/journal.pgph.0003366 (PMC11542803; doi:10.1371/journal.pgph.0003366)
Supplement: S1 File — (DOCX) [file pgph.0003366.s001.docx]

# **Reporting checklist for qualitative study.**

Based on the SRQR guidelines.

**Title** Facilitators and barriers to decision-making for hospital treatment among patients diagnosed with breast cancer in Dar es Salaam, Tanzania: A qualitative urban-based study

|  |  | Reporting Item | Page Number |
| --- | --- | --- | --- |
| **Title** |  |  |  |
|  | [#1](https://www.goodreports.org/srqr/info/#1) | Concise description of the nature and topic of the study identifying the study as qualitative or indicating the approach (e.g. ethnography, grounded theory) or data collection methods (e.g. interview, focus group) is recommended | Pg 1 |
| **Abstract** |  |  |  |
|  | [#2](https://www.goodreports.org/srqr/info/#2) | Summary of the key elements of the study using the abstract format of the intended publication; typically includes background, purpose, methods, results and conclusions | Page 1-2 (Lines 13-34) |
| **Introduction** |  |  |  |
| Problem formulation | [#3](https://www.goodreports.org/srqr/info/#3) | Description and significance of the problem / phenomenon studied: review of relevant theory and empirical work; problem statement | Page 2-4 (Lines 53-87) |
| Purpose or research question | [#4](https://www.goodreports.org/srqr/info/#4) | Purpose of the study and specific objectives or questions | Pg 4 (Lines 85-87) |
| **Methods** |  |  |  |
| Qualitative approach and research paradigm | [#5](https://www.goodreports.org/srqr/info/#5) | Qualitative approach (e.g. ethnography, grounded theory, case study, phenomenolgy, narrative research) and guiding theory if appropriate; identifying the research paradigm (e.g. postpositivist, constructivist / interpretivist) is also recommended; rationale. The rationale should briefly discuss the justification for choosing that theory, approach, method or technique rather than other options available; the assumptions and limitations implicit in those choices and how those choices influence study conclusions and transferability. As appropriate the rationale for several items might be discussed together. | Pg 4-4 (Lines 91-95) |
| Researcher characteristics and reflexivity | [#6](https://www.goodreports.org/srqr/info/#6) | Researchers' characteristics that may influence the research, including personal attributes, qualifications /experience, relationship with participants, assumptions and / or presuppositions; potential or actual interaction between researchers' characteristics and the research questions, approach, methods, results and / or transferability | Pg 5 (Lines 135-139) |
| Context | [#7](https://www.goodreports.org/srqr/info/#7) | Setting / site and salient contextual factors; rationale | Pg 4 (Lines 95-106) |
| Sampling strategy | [#8](https://www.goodreports.org/srqr/info/#8) | How and why research participants, documents, or events were selected; criteria for deciding when no further sampling was necessary (e.g. sampling saturation); rationale | Pg (Lines 108-122) |
| Ethical issues pertaining to human subjects | [#9](https://www.goodreports.org/srqr/info/#9) | Documentation of approval by an appropriate ethics review board and participant consent, or explanation for lack thereof; other confidentiality and data security issues | Pg 6 (Lines 184-194) |
| Data collection methods | [#10](https://www.goodreports.org/srqr/info/#10) | Types of data collected; details of data collection procedures including (as appropriate) start and stop dates of data collection and analysis, iterative process, triangulation of sources / methods, and modification of procedures in response to evolving study findings; rationale | Pg 5 (Lines 125) |
| Data collection instruments and technologies | [#11](https://www.goodreports.org/srqr/info/#11) | Description of instruments (e.g. interview guides, questionnaires) and devices (e.g. audio recorders) used for data collection; if / how the instruments(s) changed over the course of the study | Pg 5 (Lines 126-144) |
| Units of study | [#12](https://www.goodreports.org/srqr/info/#12) | Number and relevant characteristics of participants, documents, or events included in the study; level of participation (could be reported in results) | Pg 7-8 |
| Data processing | [#13](https://www.goodreports.org/srqr/info/#13) | Methods for processing data prior to and during analysis, including transcription, data entry, data management and security, verification of data integrity, data coding, and anonymisation / deidentification of excerpts | Pg 5 (Lines 146-151) |
| Data analysis | [#14](https://www.goodreports.org/srqr/info/#14) | Process by which inferences, themes, etc. were identified and developed, including the researchers involved in data analysis; usually references a specific paradigm or approach; rationale | Pg 5-6 (Lines 156-174) |
| Techniques to enhance trustworthiness | [#15](https://www.goodreports.org/srqr/info/#15) | Techniques to enhance trustworthiness and credibility of data analysis (e.g. member checking, audit trail, triangulation); rationale | Pg 6(lines 176-164) |
| **Results/findings** |  |  |  |
| Syntheses and interpretation | [#16](https://www.goodreports.org/srqr/info/#16) | Main findings (e.g. interpretations, inferences, and themes); might include the development of a theory or model, or integration with prior research or theory | Pg 8-13 (Line 206-383) |
| Links to empirical data | [#17](https://www.goodreports.org/srqr/info/#17) | Evidence (e.g. quotes, field notes, text excerpts, photographs) to substantiate analytic findings | Pg 8-13 (Line 206-383) |
| **Discussion** |  |  |  |
| Intergration with prior work, implications, transferability and contribution(s) to the field | [#18](https://www.goodreports.org/srqr/info/#18) | Short summary of main findings; explanation of how findings and conclusions connect to, support, elaborate on, or challenge conclusions of earlier scholarship; discussion of scope of application / generalizability; identification of unique contributions(s) to scholarship in a discipline or field | Pg 14-17 (Lines 384-474) |
| Limitations | [#19](https://www.goodreports.org/srqr/info/#19) | Trustworthiness and limitations of findings | Pg 17(Lines 476-485) |
| **Other** |  |  |  |
| Conflicts of interest | [#20](https://www.goodreports.org/srqr/info/#20) | Potential sources of influence of perceived influence on study conduct and conclusions; how these were managed | Pg 18 (Lines 498-499) |
| Funding | [#21](https://www.goodreports.org/srqr/info/#21) | Sources of funding and other support; role of funders in data collection, interpretation and reporting | Pg 18(Line 501-504) |

None The SRQR checklist is distributed with permission of Wolters Kluwer © 2014 by the Association of American Medical Colleges. This checklist can be completed online using <https://www.goodreports.org/>, a tool made by the [EQUATOR Network](https://www.equator-network.org) in collaboration with [Penelope.ai](https://www.penelope.ai)
