# Supplementary material for: Facilitators and barriers to decision-making for hospital treatment among patients diagnosed with breast cancer in Dar es Salaam, Tanzania: A qualitative urban-based study
Source: PLOS Glob Public Health. 2024 Nov 7;4(11):e0003366. doi: 10.1371/journal.pgph.0003366 (PMC11542803; doi:10.1371/journal.pgph.0003366)
Supplement: S2 File — (DOCX) [file pgph.0003366.s002.docx]

**Interview guide**

**Title: Facilitators and barriers to decision-making for hospital treatment among patients diagnosed with breast cancer in Dar es Salaam, Tanzania: A qualitative urban-based study**

Interview Site ………………………………………………………...…………

Interview Number ………………………………………………………………

Date ……………………………………………………………………….……

Start time ………………………………………………………………………..

End time …………………………………………………………………………

Participant Demographic Information

Age…. …………………………………………………………………………

Occupation ………………………………………………………….…………

Stage of Cancer………………………………………………………………...

Education …………………………………………………….………….…….

Religion ………………………………………………………………….…….

Type of treatment………………………………………………………………

Marital status…………………………………………………………………...

**Introduction:**

Thank you for agreeing to participate in the research. This interview will take 30 minutes to 45 minutes and it will be recorded. I would like to ask your permission for taping/record the session because I do not want to miss any of your ideas and suggestions. Because we are recording, please speak up so that we do not miss your useful ideas. I would like to assure you that every information that you provide will be confidential. Any information we use from your interview will be combined with information from other participants who will be participated in this research interview. I assure you of the confidentiality of the information you provide for this research. Do you have any questions before we start the interview?

**Guiding Questions**

Now we are going to discuss your treatment decision-making.

1. How did you feel when you were first diagnosed with breast cancer? Probe on personal perception, cause, treatability, etc)
2. Did you think of seeking alternative treatment? (If yes, probe on reasons, types of alternative treatment sought(traditional medicine, spiritual healing, Chinese medicine, the outcome of treatment etc)
3. What influenced you to seek hospital treatment? (Probe on self, spouse, parents, relatives, healthcare providers, etc)
4. What factors did you consider before making the decision for hospital treatment?

Probe on

1. Personal, social, economic, institutional, etc)
2. Why do you think the factors you mentioned are important in your treatment decision-making?
3. What information were you given before making the treatment decision? (Probe on treatment options, benefits and risk of cancer treatments, the outcome of treatment etc )
4. How free were you in making decision for your treatment? (Probe on freedom of choice, right to refuse treatment, etc )
5. What are your views regarding your decision to receive treatment here at ORCI? (beneficial, non-beneficial, guilty of decision, etc)
6. What challenges did you get during the decision-making for treatment? (Probe on moral, religious, social, financial etc)
7. What makes you continue with hospital treatment(Probe on interaction with healthcare providers, social or family support, diagnostic and treatment services, the influence of significant others, etc)
8. What would make you discontinue hospital treatment or hospital services? (probe on influence of significant others, poor interaction with health care providers, stage of cancer, personal factors, stigma, economic factors, family factors)
9. What would you suggest to a person diagnosed with cancer regarding treatment decision-making?
10. Is there anything else that you would like to share with me about what we have just discussed or anything else we have not discussed?

Closure of the interview: Is there anything else that you would like to share with me about what we have just discussed?

Thank You for Your Participation
